# Umbilical cord blood derived cellular therapy: advances in clinical development

**DOI:** 10.3389/fonc.2023.1167266

**Published:** 2023-05-18

**Authors:** Jiasheng Wang, Leland Metheny

**Affiliations:** Department of Hematology/Oncology, University Hospitals Seidman Cancer Center, Cleveland, OH, United States

**Keywords:** cord blood, chimeric antigen receptor, hematopoietic stem cell transplantation, NK-cells, mesenchymal stem cells

## Abstract

While cord blood (CB) is primarily utilized in allogeneic hematopoietic cell transplantation (HCT), the development of novel cell therapy products from CB is a growing and developing field. Compared to adult blood, CB is characterized by a higher percentage of hematopoietic stem cells (HSCs) and progenitor cells, less mature immune cells that retain a high capacity of proliferation, and stronger immune tolerance that requires less stringent HLA-matching when used in the allogenic setting. Given that CB is an FDA regulated product and along with its unique cellular composition, CB lends itself as a readily available and safe starting material for the development of off-the-shelf cell therapies. Moreover, non-hematologic cells such as mesenchymal stem cell (MSCs) residing in CB or CB tissue also have potential in regenerative medicine and inflammatory and autoimmune conditions. In this review, we will focus on recent clinical development on CB-derived cellular therapies in the field of oncology, including T-cell therapies such as chimeric antigen receptor (CAR) T-cells, regulatory T-cells, and virus-specific T-cells; NK-cell therapies, such as NK cell engagers and CAR NK-cells; CB-HCT and various modifications; as well as applications of MSCs in HCT.

## 1 Introduction

The umbilical cord (UC) connects the fetus to the placenta for nutrient uptake, waste elimination, and gas exchange. The UC contains one vein, two arteries, and is protected by a surrounding gelatinous substance called Wharton’s jelly. At term, the UC has a mean length of 50cm, a mean diameter of 14mm, an approximate weight of 40g, and together with the placenta contains 50 to 200mL of cord blood (CB) (1). Compared to adult blood, CB contains a higher percentage of hematopoietic stem cells (HSCs) (2); moreover, the immune cells, such as T-cells and NK-cells, are phenotypically less mature (3, 4) These CB-specific features allow for more robust *ex vivo* and *in vivo* hematopoietic expansions and greater immune tolerance when used as a source for allogeneic cellular therapies. Apart from blood cells, nonhematopoietic progenitor cells such as mesenchymal stem cells (MSCs) can also be readily obtained from CB or CB tissue compartments such as Wharton’s jelly, which have great potential in regenerative medicine and immune modulation.

Following the first successful CB hematopoietic cell transplantation (CB-HCT) in 1988 and the establishment of the first public CB bank in New York in 1991 (5), more than 100 public CB banks were established worldwide with millions of CB units donated altruistically, greatly expanding the availability of CB (6). Moreover, it was estimated that more than 4 million units are being cryopreserved in private CB banks (7). Additionally, CB is an FDA regulated product that adheres to regulatory requirements. Therefore, the CB banks can provide readily available, high quality, and immunologically compatible hematopoietic cells to the majority of the population (8). CB is a particularly important cell source for patients who are racial or ethnic minorities, where matched adult donors are lacking in the registry (9). Although CB-HCT remains the main application of public CB banks, recent progress in cellular therapy has shown great potential in other CB-derived cellular products. Therefore, CB banks are an important resource to quickly upscale CB-derived cellular therapy. In addition to public cord blood banks, private cord blood banks offer the option of storing and using autologous cells for future use. However, preserving cord blood cells at birth for personal use poses several challenges. Firstly, the likelihood of needing CB-HCT, which is currently the only FDA-approved use for CB cells, is very low for people without a family history of blood disorders, estimated to be around 1 in 20,000 in their lifetime (10). Moreover, autologous CB-HCT is less effective than allogeneic CB-HCT in treating malignancies due to the absence of GvL effect. Thirdly, the quantities of stored single unit autologous cells may not be sufficient for cellular therapy. Finally, the impact of prolonged cryopreservation on the functionality of CB cells remains unclear. While the transplantation outcomes appear unaffected when CB cells were cryopreserved for up to 10 years, longer preservation may affect the viability of CB cells (11).

CB-derived cellular therapy harbors certain limitations. First, biologically, CB might not be the ideal source for all cellular therapies. For example, virus-specific T-cells (VSTs) have been used in severely immunosuppressed patients with resistant viral infections. However, given the naïve nature of CB, it lacks virus-experienced T-cells to manufacture VSTs (12). Second, practically, CB is limited by the small volume and total amount of hematopoietic cells. Therefore, *ex vivo* expansion may be required prior to adoptive cellular transfer. Moreover, good manufacturing practice (GMP)-compliant expansion protocols need to be established for each type of cellular therapy (13). Third, acquisition, processing, and storage of CB are expensive, which may result in low utilization of CB units from CB banks (7). Therefore, the high cost of each CB unit may translate into expensive CB-derived cellular products. Indeed, the current cost of a CB unit for HCT in developed countries ranges from thirty- to sixty-thousand US dollars (14).

**Figure 1:**
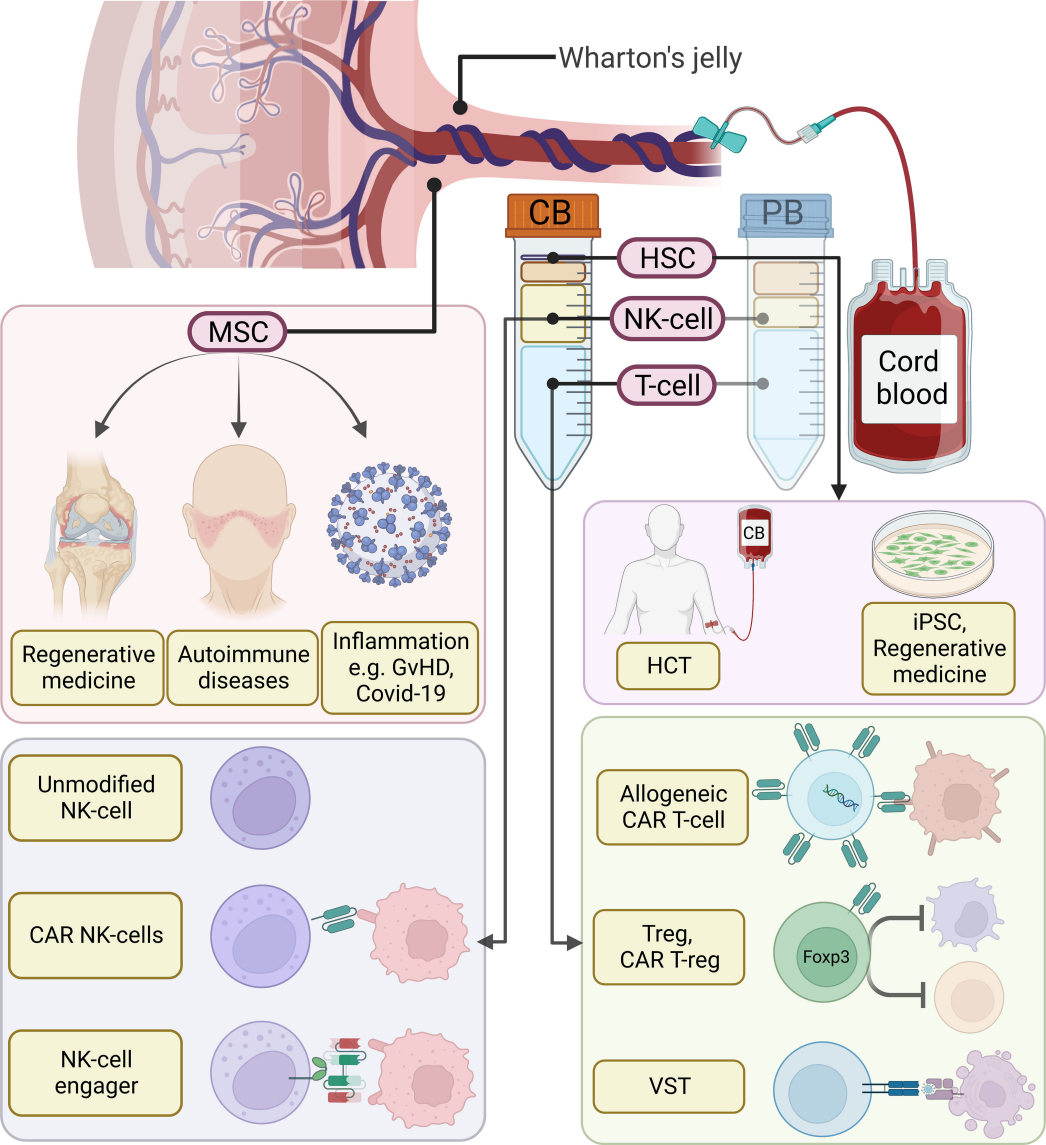
Overview of cord blood (CB) derived cellular therapies. Compared to adult peripheral blood (PB), CB is characterized by a higher percentage of NK-cells and the presence of hematopoietic stem cells (HSCs). Moreover, blood cells in CB are more naïve and harbor greater proliferation potential. Among them, HSCs can be used in hematopoietic stem cell transplantation (HCT) or as a source for induced pluripotent stem cells (iPSCs) and regenerative medicine. T-cells can be utilized to generate allogeneic chimeric antigen receptor (CAR) T-cells, regulatory T-cells (Tregs), CAR T-regs, or virus-specific T-cells (VSTs). CB-derived NK-cells have been shown to be a great source for allogeneic NK-cell infusion, CAR NK-cells, as well as pre-activated NK-cells pre-complexed with NK-cell engagers. In addition to CB-derived blood cells, nonhematologic cells in the Wharton’s jelly, such as mesenchymal stem cells (MSCs), process regenerative and immunomodulatory effects. MSCs can be used in tissue engineering, autoimmune diseases, as well as inflammatory diseases.

In this review, we will discuss cellular therapies utilizing different hematologic components of CB, including T-cells, NK-cells, HSCs, and hematopoietic progenitor cells, as well as non-hematologic components such as MSCs (**Figure 1**). This review will focus on their applications in oncology based on clinical evidence from published clinical trials, and discuss advantages, challenges, and limitations of each CB-derived cellular product (**Table 1**)

**Table 1:** Overview of advantages and challenges of cord blood-derived cellular therapies .

Cellular component	Product or indication	Clinical development phase	Advantages	Challenges	Future development
T-cell	CAR T	Ongoing Phase I	• More naïve T-cell phenotype, may translate into better *in vivo* expansion and persistence (15) • Ability to generate an off-the-shelf bank matching HLA of most population, avoiding GvH and HvG effects without genome editing (16) • Able to incorporate CB-HCT before or after CAR T-cell therapy (17) • CB stored at birth can be used autologously avoiding contamination from T-cell leukemia	• Limited starting material • Lack proof-of-concept early phase clinical trial	• Proof-of-concept clinical trials with HLA-matched CB-derived CAR T-cell product demonstrating *in vivo* expansion and persistence • Use techniques such as iPSC to generate bulks of T-cells
	Treg	Phase I/II, preclinical for CAR-Tregs	• Higher percentage of Tregs in CB (18) • Allogeneic and off-the-shelf ability given HLA-mismatch tolerance	• Limited starting material • Limited persistence in blood, may need repeated infusion (19)	• CAR-Tregs to target specific antigen • Use techniques such as iPSC to generate bulks of Tregs
	VST	Phase I/II	• Able to utilize 20% of CB units for CB-HCT to generate VST (12)	• Naïve CB T-cells lacking virus-experienced T-cells • Prolonged production time due to limited starting material (12)	• Allogeneic PB-derived VST likely a better source than CB-derived VST
NK-cell	NK infusion	Phase I/II	• Higher percentage of NK cells in CB (2) • Improved persistence of HLA-matched CB-derived NK cells • Bulk production of HLA-matched CB-derived NK cells through induced differentiation of HSCs (20)	• Limited activity in early phase trials (21) • Reduced cytotoxicity compared to PB NK cells if without cytokine stimulation (22) • Lack of *in vivo* expansion and persistence (23) • Poor intra-tumoral infiltration (24) • Cryopreservation of CB can damage NK cell cytotoxicity (25)	• Combine other agents such as interleukin, TGFβ receptor inhibitor to improve efficacy • Genetically engineered NK cells, such as self-IL-15 secretion, to improve expansion and persistence
	CAR NK	Phase I/II	• Favorable safety profile without risk of GvHD, CRS, or ICANS (26) • Enhanced killing through both CAR and innate receptor, theoretically reducing CAR antigen escape (27) • Higher percentage of phenotypically naïve NK cells in CB promoting *in vivo* expansion (28) • Lack of innate inhibitory NK signal given the allogeneic nature • As a CB-derived product, readily available cord banks and high proliferative potential	• Requires *ex vivo* expansion • Limited *in vivo* persistence, leading to early relapse (26) • Tumor suppressive environment hindering efficacy (29) • Feasibility of large-scale manufacturing	• Genetically engineered NK cells to improve expansion and persistence • Approaches to tackle trogocytosis
	NKCE+NK	Phase I/II	• Proven off-the-shelf ability (30) • Favorable safety profile without risk of GvHD, CRS, or ICANS (30) • Relatively lower cost than genetically engineered cells • Multiple indications with the same platform	• Limited efficacy if without co-infusion of preactivated NK cells; cumbersome to administer (31)	• Confirmation trials with a larger population • Expansion of indications in other types of malignancies
Stem cell	CB-HCT	FDA-approved	• Readily available source • Less stringent HLA-matching requirement (32) • Less chronic GvHD (33) • FDA-approved *ex vivo* expanded CB products enhancing engraftment (34)	• Low stem cell dose • Slow engraftment, leading to increased transplant-related morbidity and mortality (35) • Increased resource utilization • Extra processing time and cost for *ex vivo* expanded CB products	• Increased utilization of *ex vivo* expanded CB products, such as omidubicel, in the clinical setting • Further development of *ex vivo* expanded CB products • Reducing cost for *ex vivo* expanded CB products
MSC	GvHD	Phase II	• Easily obtained source • Good safety profile (36) • Efficacy in early phase trials (37–39) • Off-the-shelf ability	• A phase III trial of BM-derived MSCs failed to show improved efficacy over existing care (40)	• Carefully designed phase III trials in later lines of therapy for GvHD • Efficacy in chronic GvHD • Identify markers to predict responders

CAR, chimeric antigen receptor; HLA, human leukocyte antigen; GvH, graft-versus-host; HvG, host-versus-graft; CB, cord blood; HCT, hematopoietic cell transplantation; iPSC, induced pluripotent stem cells; VST, virus-specific T-cells; PB, peripheral blood; HSC, hematopoietic stem cells; CRS, cytokine release syndrome; ICANS, immune effector cell-associated neurotoxicity syndrome; NKCE, natural killer cell engager; ARDS, acute respiratory distress syndrome; BM, bone marrow.

## 2 Cord blood-derived T-cell therapies

CB T-cells differ in composition and functional properties when compared to peripheral blood (PB) T-cells. Close to 90% of T-cells in the CB are naïve T-cells since they have not encountered any foreign antigens (3). Moreover, CB harbors a higher percentage of CD8+CCR7+ T-central memory (T_CM_) cells, which mediates enhanced antitumor responses through increased tumor-homing and rapid gain of cytotoxic function, making it an excellent source for adoptive T-cell therapies (41, 42). However, CB T-cells are not simply immature versions of adult T-cells, but a distinct population with different gene expression profile and functional properties (43). They are more tolerant-prone and poised for rapid effector differentiation (44), which are ideally suited for allogeneic T-cell therapies.

T-cell therapies in CB can be broadly divided into two categories, one involves isolation and expansion of existing T-cells (Tregs, or virus-specific or tumor antigen-specific cytotoxic T-cells), and the other involves genetic engineering of T-cells. The latter, which in the early years utilized T-cell receptor (TCR) engineering, has been associated with suboptimal clinical efficacy and considerable toxicities (45). The recent years have witnessed a rapid development of chimeric antigen receptor (CAR) T-cell therapy, which has demonstrated superior clinical efficacy especially in B-cell malignancies (46), making it an ideal platform for CB-based T-cell therapies.

### 2.1 Allogeneic CAR T-cell therapy

CAR T-cell therapy has revolutionized the treatment of refractory/relapsed B-cell malignancies. However, current FDA-approved CAR T-cells are all autologous products derived from patients, limited by long production time, chance of production failure, product variability, possible contamination by tumor cells, and high production cost. Therefore, “off-the-shelf” allogeneic CAR T-cells have attracted great attention in recent years, albeit encountering many challenges, such as graft (CAR T)-*versus*-host (GvH) effects due to Human Leukocyte Antigens (HLA) mismatch, and lack of *in vivo* expansion and persistence due to host-*versus*-graft (HvG) effects (47). Using CB as the source for allogeneic CAR T-cells may overcome some of the challenges and offer unique advantages over PB from healthy donors. First, CB CAR T-cells retained a less differentiated phenotype (such as T_SCM_ and T_CM_) than PB CAR T-cells (15), which could translate into a longer persistence of CAR T-cells. Second, CB T-cells have been known to have greater immune tolerance to HLA mismatch (16). In CB HCT, matching for alleles HLA-A, HLA-B and HLA-DR (i.e. ≥ 4 of 6 HLA match) is sufficient to reduce the incidence of graft-*versus*-host disease (GvHD) to a low level (48). Therefore, it has been demonstrated that an CB bank of only 150 selected samples could provide full matches (6 of 6 HLA loci) for 93% of the UK population (49); if partial mismatch is allowed (4 or 5 of 6 HLA loci), the required number can be further reduced. Therefore, it is possible to generate an “off-the-shelf” allogeneic CB-derived CAR T-cell bank that can be readily used for most of the population with minimal GvH effects. On the other hand, to reduce GvH effects in PB-derived CAR T-cells, T-cell receptor (TCR) knockout using genome editing is required, for which the long-term safety is still unknown (50). Another advantage of CB-derived CAR T-cell therapy is the ease to incorporate consolidative CB HCT after CB CAR T-cell therapy, which might provide improved progression-free survival (17).

Several preclinical studies have demonstrated the feasibility of manufacturing CAR T-cells from CB (15, 41, 51). In an earlier study by Pegram et al., banked CB T-cells were expanded with IL-12/IL-15 stimulation, and transduced with anti-CD19 and IL-12 secreting modules (51). The CAR T-cells retained a central memory-effector phenotype, and demonstrated activity in a B-cell acute lymphoblastic leukemia mouse model. In a study by Cael et al., fresh or thawed CB T-cells were transduced by lentivirus encoding CD123-CAR to target blastic plasmacytoid dendritic cell neoplasm (BPDCN) mouse models (15). They found that CB CAR T-cells exhibited potent anti-tumor activities while retaining a less differentiated profile than PB CAR T-cells; moreover, fresh or cryopreserved CB CAR T-cells demonstrated similar functionalities. In another study by Liu et al., cryopreserved, CB-derived, CD19-targeting CAR T-cells had more T_N_ and T_CM_ than PB CAR T-cells derived from the patients, and harbored fewer T-cell exhaustion markers, leading to a better antitumor efficacy and longer tumor-suppressive effects (41).

However, allogeneic use of CB as an “off-the-shelf” product in the clinical setting is still lacking. A search of ClinicalTrials.gov in December 2022 returned only one ongoing clinical trial (NCT03881774). Indeed, several limitations of CB-derived CAR T-cells need be addressed before broader clinical applications. First, like other allogeneic CAR T-cell therapies, solutions need be developed to tackle GvH and HvG effects. Although TCR knockout has been effective to reduce GvH in PB-derived (52) and CB-derived (53) CAR T-cells, it is possible to avoid genome editing, as mentioned above, by obtaining an HLA-matched CB unit from a limited and highly selected CB bank as the cell source for CAR T-cells (49). However, proof-of-concept clinical studies are lacking at this point. Second, due to limited availability of selected CB units, techniques such as induced pluripotent stem cells (iPSCs) (54) or directed differentiation of hematopoietic stem cells (55) are required to produce the bulk population of T-cells. However, further developments of these methods are still needed to generate a self-renewing and clinically applicable source of T-cells.

### 2.2 Regulatory T-cells (Tregs)

Tregs are a subset of natural occurring CD4+ T-cells that suppresses the activation and expansion of overactive lymphocytes, thus preventing excessive immune activation and autoimmunity. While Tregs comprise less than 3% of mononuclear cells in the PB, CB contains a high proportion of circulating natural Tregs, obviating multiple selection processes to reach the required purity of Tregs (18). Moreover, compared to PB-derived Tregs, CB-derived Tregs have greater post-expansion purity, FOXP3 stability, and proliferative capacity (56). Another advantage is the potential of CB units as an allogeneic source of Tregs due to great tolerability of HLA-mismatch. Adoptive cellular therapy using Tregs can be divided into two categories: selection and expansion of natural occurring polyclonal Tregs, and manufacturing antigen specific Tregs using TCR or CAR gene transfer.

Polyclonal Tregs have been reported in human trials in treating autoimmune diseases such as multiple sclerosis (57) and type 1 diabetes mellitus (58, 59), preventing rejection and reducing needs for immunosuppressant agents after solid-organ transplantation (20, 60, 61), and preventing and treating GvHD after HCT (19, 62–64). While most of the aforementioned studies used autologous PB mononuclear cells (PBMC) as the source of Treg, in a study by Brunstein et al., 11 patients undergoing CB-HCT received CB-derived, HLA-matched Tregs from a cord bank; they observed very low incidence of Grade II-IV acute GvHD of only 9%, and none developed chronic GvHD (19). However, although Tregs have shown a favorable safety profile and early signs of clinical efficacy, several challenges must be overcome before broader applications. The first challenge is to determine the timing and frequency of administration. Although in some studies Tregs can still be detected in the PB one year after administration (59), other studies have shown the persistence of only 14 days (19). Therefore, for conditions like autoimmune disease or solid-organ transplantation where persistent immunosuppression is required, it is unknown whether and when Treg products should be re-dosed after the initial infusion. Indeed, in the landmark ONE trial among patients with kidney transplantation, after a single dose of autologous Treg infusion, immunosuppressants were able to be tapered but not completely weaned off (60). Second, due to limited volume of CB, it is not uncommon to fail to achieve the targeted cell dose, which occurres in a frequency of more than 10% in previous studies (18, 19). Therefore, a better understanding of Treg biology and improvements of manufacturing process are needed for future larger trials. While identifying new Treg markers can simplify Treg selection and purification, techniques such as 3D culture, automated bioreactors, or robotic platforms may aid in the large-scale production of lineage-stable CB-derived Treg cells.

While polyclonal Tregs may be suitable for the treatment of autoimmune conditions where a variety of tissues are targeted, antigen-specific Tregs may be particularly useful in situations where the disease-relevant antigens are known or limited tissues are involved (65). Through engineered antigen-specific receptors (CARs or TCRs), Tregs have enhanced trafficking and targeting to specific microenvironment. With the success of CD19-targeting CAR T-cell therapy, CAR-Tregs have attracted great attention in recent years, albeit current studies have been limited to the preclinical phase. Major conditions being studied include HCT/solid organ transplantation (66), type 1 diabetes (67), inflammatory bowel disease (68), and hemophilia (69), targeting HLA-A2, insulin/HiP2, carcinoembryonic antigen (CEA), and factor VIII, respectively. A search of ClinicalTrials.gov in December 2022 showed two ongoing clinical trials (NCT04817774 and NCT05234190), both using autologous, HLA-A2 targeting CAR Tregs to prevent rejection in solid-organ transplantation recipients who are HLA-A2 negative receiving an A2 positive graft. Hurdles for manufacturing autologous CAR Tregs for clinical use include prolonged production time, contamination of conventional T-cells, and higher chance of production failure due to limited amount in the PB (70). Moreover, in a population often receiving high dose immunosuppressants, the function of Tregs might be impaired. Therefore, “Off-the-shelf” allogeneic CAR Tregs products, such as those manufactured from CB, are promising alternatives. However, further studies are needed to demonstrate their safety and efficacy.

### 2.3 Virus-specific T-cells (VSTs)

Over the past three decades, adoptive transfer of VSTs has been proven to be safe and effective for the treatment of refractory viral infections in patients with severely compromised immune system, such as those underwent HCT/solid-organ transplantation (71) or with primary immunodeficiency disorders (72). Moreover, the indications of VSTs have been extended to viral-associated malignancies such as EBV-positive post-transplantation lymphoma (73–75). These VSTs are usually organ or stem-cell donor derived, either through *ex vivo* stimulation and expansion, or direct sorting and selection from apheresis products of seropositive donors (76). True allogeneic VSTs obtained from healthy third-party donors have been increasingly reported in recent years. Even with low level HLA match (1 or 2 out of 6 or 8), studies have shown excellent clinical efficacy without causing significant GvHD (77–79). The extremely low rates of acute GvHD (<10%) are probably due to the fact that VSTs are largely central or effector memory T cells that are less likely to be alloreactive (80). However, studies have shown that persistence of third-party allogenic VSTs was only 12 weeks (78), which might not be enough to cover the whole immune reconstitution phase. Therefore, future studies are needed to examine the necessity and timing of redosing or investigate methods to promote allogeneic VST’s persistence.

However, CB has several major limitations as a source for VSTs. First, CB contains predominantly naïve T-cells lacking virus-experienced T-cells, which makes direct sorting and selection difficult. Second, a single CB unit has a small volume; with the inability to procure further donations, this leads to prolonged expansion and production time with high chances of production failure. However, for patients undergoing CB HCT, CB becomes the only available source for autologous VSTs. Abraham et al. reported the use of 20% fraction of CB units that were reserved prior to the stem cell infusion, and they used them to manufacture CB-derived multivirus-specific T-cells in pediatric patients (12). Using *ex vivo* antigen stimulation, production was successful in 18 of the 21 pediatric patients. However, the median production time was 52.5 days, which remains the major obstacle for widespread application given the urgency of antiviral treatment. Further development has resulted in reduced production time, but it could still take around 30 days (81). Moreover, the expanded cell dose is likely insufficient for adult patients. Therefore, although it is feasible to produce VSTs from CB, challenges remain; given the efficacy and safety of allogeneic PBMC-derived VSTs, they will likely come out as a better option for patients undergoing CB HCT complicated by refractory viral infections.

### 2.4 Other T-cell therapies

Autologous HCT using stored self CB have been reported (82). Therefore, it is also possible to manufacture autologous CAR T-cells from preserved CB at birth. This may be particularly useful for patients with T-cell leukemia where it is vital to avoid CAR T-cell product contamination from circulating leukemic T-cells.

γδ T-cells is a unique subpopulation of T-cells that function in between innate and adaptive immunity – they mediate cytotoxicity through antigen-pattern recognition independent of MHC presentation (83). Over the past two decades, adoptive transfer of PB-derived autologous γδ T-cells have been tested in a wide range of malignancies without good clinical response (84). Interestingly, given the HLA-independent killing, allogenic γδ T-cells have been proven to be safe with a low risk of GvHD. Therefore, repeated infusion of healthy donor derived allogeneic γδ T-cells has become possible. Indeed, two recent studies showed improved clinical efficacy with repeated γδ T-cells infusion in patients with late-stage malignancies (85, 86). Furthermore, targeted therapy using CAR-engineered γδ T-cells have attracted great attention, with a recent phase I study showed promising safety and efficacy (87). On the other hand, CB has several disadvantages as the source for γδ T-cell therapies, including low frequency of γδ T-cells (<1%) and lack of the functional subset of γδ T-cells (88). Although successful expansion of CB-derived γδ T-cells has been reported (89, 90), its clinical usage remains to be defined, given that PB-derived γδ T-cells can be more easily manufactured.

A broader application of CAR T-cell therapy in solid tumors is limited due to the lack of tumor-specific surface antigens. However, frequently mutated intracellular protein can be presented to the cell surface through coupling with MHC I molecules, forming neoantigens that can be recognized by TCRs; this provides an opportunity for genetically engineered TCR-T cell therapy. A recent study using autologous T cells transduced with TCRs recognizing the mutant KRAS G12D-MHC I complex showed a partial response in a patient with metastatic pancreatic cancer (91). CB-derived TCR T-cell therapy has remained in the pre-clinical phase (92).

Lastly, tumor antigen-specific cytotoxic lymphocytes (CTLs) can be selectively expanded (93) or primed (94) using techniques similar to manufacturing VSTs. Compared to CTLs obtained from PB, CB-derived CTLs showed higher proliferative and cytotoxic activities in samples obtained from patients (95). Antigen-specific CTLs might be useful in patients who have received CB HCT to prevent or treat cancer relapse, in a role similar to donor lymphocyte infusion (DLI). However, research in this field is extremely limited.

## 3 CB-Derived NK-cell therapies

NK cells are part of the innate immune system that target malignant or virus-infected cells. Unlike T cells, NK cells lack specificity to a certain antigen. Rather, their activation is based on a “signal threshold” from the integration of inhibitory and activating receptors – bindings between NK inhibitory receptors and target cell HLA molecules provide the inhibitory signals, while the engagements of NK activating receptors with stress-induced self-ligands provide the activating signals (96). When HLA expression is decreased due to infection or malignant transformation, or when NK cells are used in the allogeneic setting, the inhibitory signals are weakened; along with increased activating signals, NK cells are activated.

While NK cells are the main effector for the early graft-*versus*-leukemia reactions, they do not directly cause GvHD even with HLA-mismatch (97), making them an ideal source for allogeneic adoptive cell therapy (98). NK cells are enriched in CB comprising up to 30%, whereas in the PB they only constitute 10% of lymphocytes (2). Furthermore, it has been observed that NK cells reconstitute more rapidly after CB HCT than PB HCT (99), which is likely due to better homing capability of HCB NK cells to the bone marrow (4) and the presence of a unique progenitor-like subpopulation (28). In addition, much like CB T cells, CB NK cells are less mature than PB NK cells, as characterized by the reduced expression of activation receptors (4). While this leads to reduced cytotoxicity compared to PB NK cells, their activities can be enhanced with cytokine stimulation (22). Therefore, allogeneic NK cell therapies especially using CB as the source have attracted great attention in recent years. However, limited persistence and modest antitumor activities remain the two biggest barriers in the field of NK cell therapy.

### 3.1 Allogeneic NK cell infusion from a CB source

Earlier clinical trials using unmodified autologous NK cell infusion demonstrated only modest success (21). Advancement in allogeneic HCT has helped us better understand the physiology of NK cells – the mismatch between NK receptors and ligands are the key for the graft-*versus*-leukemia effect. In the past two decades, haploidentical NK cell therapies tested in patients with hematologic malignancies with or without prior HCT, have demonstrated feasibility, safety, and signals of efficacy (100–108). However, most of the studies used PB from healthy donors as the source for NK cells; clinical studies on infusion of unmodified NK cells from a CB source are scarce. Shah et al. reported the infusion of *ex vivo* expanded, 4/6 HLA-matched, cryopreserved CB-derived NK cells to patients with multiple myeloma, in between high-dose melphalan conditioning and autologous HCT (109). They observed no dose-limiting toxicities. Recently, our group reported the use of HLA-mismatched NK cells in patients with refractory malignancies; while we observed a good safety profile without GVHD, NK cells only persisted less than 4 weeks (97). Therefore, HLA-match or partial match is likely not required from the safety standpoint, but it may play a role in preventing allorejection and ensuring long-term persistence.

In addition to the “isolation-expansion” approach described above, NK cells can also be obtained through differentiation of hematopoietic stem and progenitor cells (HSPCs). This approach has been shown to result in a higher purity of NK cells with an improved bone marrow homing capacity (110). Dolstra et al. reported the first-in-human use of isolating CD34+ HSPCs from partially HLA-matched CB units, and inducing *ex vivo* NK cell differentiation by applying IL-2 and IL-15 (111). The final product was infused in 10 elderly patients with acute myeloid leukemia in remission, and they observed a good safety profile with preliminary efficacy as 2 of the 4 patients with positive measurable residual disease (MRD) turned negative. Nevertheless, larger and comparative studies are needed to determine whether induced NK cells have better clinical outcomes than isolated NK cells.

### 3.2 CB-derived NK cells complexed with NK cell engagers

Due to the modest antitumor activities of unmodified NK cell infusion, various strategies have been developed in the past few years to improve NK cell recognition and activation, including overexpressing chemokines or chemokine receptors to enhance NK cell tumor infiltration (112, 113), concurrent use of immune checkpoint inhibitors to reverse NK cell exhaustion (114), blocking the immunosuppressive TGF-β signaling from the TME (115), and promoting NK cell tumor-specific engagement through NK cell engagers (NKCEs) (31, 116–119). Of note, almost all have remained in the preclinical/early clinical phase.

Among them, NKCEs have received most attention in recent years due to excellent preclinical results, with multiple ongoing early phase clinical trials in various types of malignancies (**Table 2**). NK cells exert their cytotoxic activities through three main mechanisms: direct killing by perforin/granzyme release, cytokine secretion, and antibody-dependent cell-mediated cytotoxicity (ADCC) though Fc-region binding receptor CD16 (FcγRIIA). The third mechanism provides a unique opportunity to pair monoclonal antibodies and NK cells to enhance their anti-tumor activities, prompting the development of NKCEs. Depending on the number of modules, NKCEs can be bispecific killer cell engagers (BiKEs) targeting CD16 expressed on NK cells and antigens of interest on tumor cells, or trispecific killer cell engagers (TriKEs), where an additional IL-15 module was added to promote NK cell expansion and persistence (31). Although NKCEs can be given as a monotherapy, the response rate was suboptimal. For example, when an anti-CD16/anti-CD30 bispecific NKCE, AFM13, was administered in patients with relapsed/refractory Hodgkin lymphoma, only 16.7% response rate was observed (120). However, a preclinical study by Kerbauy et al. combined AFM 13 with cytokine-induced memory-like NK cells and infused them in a pre-complexed manner (117). The authors found the combination of NKCE and pre-activated NK cells significantly improved the efficacy against CD30+ cancers compared to using either NKCE or NK cells alone. Moreover, when CB-derived NK cells were pre-activated by a set of cytokines and complexed with NKCE, they mounted an augmented response against CD30+ cancers (117). Currently, a phase I/II clinical trial (NCT04074746) is actively investigating this approach using CB-derived NK cells in patients with CD30+ T-cell lymphoma and Hodgkin lymphoma. Preliminary results of 30 patients with relapsed/refractory CD30+ lymphoma showed that this approach was safe – without infusion reaction, CRS, ICANS, or GvHD of any grade – and demonstrated an impressive efficacy with an 97% overall response rate and 63% complete response rate (30).

**Table 2:** Ongoing trials of cord blood-derived CAR NK-cells or NK-cells pre-complexed with NK-cell engagers (NKCEs).

Reference	Sponsor	Phase of trial	Product type	Product design	Indication	Dose of NK cells	Pre-conditioning prior to infusion	Safety outcome	Efficacy outcome
NCT05472558	Second Affiliated Hospital, Zhejiang University, China	Phase I recruiting (n=48)	CAR NK	Anti-CD19	R/R B-NHL	2×10^6^/kg, 4×10^6^/kg, 8×10^6^/kg	N/A	N/A	N/A
NCT04796675	Wuhan Union Hospital, China	Phase I recruiting (n=27)	CAR NK	Anti-CD19, IL-15 co-expression	R/R B-NHL	0.1×10^6^/kg, 1×10^6^/kg, 10×10^6^/kg	Fludarabine (30mg/kg), Cyclophosphamide (300mg/kg)	N/A	N/A
NCT05247957	Lu Daopei Hospital, China	Phase I recruiting (n=9)	CAR NK	Anti-NKG2D ligand	R/R AML	2×10^6^/kg, 6×10^6^/kg, 18×10^6^/kg	N/A	N/A	N/A
NCT05008536	Xinqiao Hospital, China	Phase I recruiting (n=27)	CAR NK	Anti-BCMA	R/R MM	1×10^6^/kg, 6×10^6^/kg, 18×10^6^/kg	Fludarabine (30mg/kg), Cyclophosphamide (300mg/kg)	N/A	N/A
NCT05092451	MD Anderson	Phase I/II recruiting (n=94)	CAR NK	Anti-CD70, IL-15 co-expression	CD70-expressing R/R hematologic malignancies	N/A	Fludarabine, Cyclophosphamide	N/A	N/A
NCT05110742	MD Anderson	Phase I/II recruiting (n=48)	CAR NK	Anti-CD5, IL-15 co-expression	CD5-expressing R/R hematologic malignancies	1×10^7^, 1×10^8^, 1×10^9^, 1×10^10^	Fludarabine, Cyclophosphamide	N/A	N/A
NCT03056339 (26)	MD Anderson	Phase I published (n=11)	CAR NK	Anti-CD19, IL-15 co-expression	R/R B-NHL	0.1×10^6^/kg, 1×10^6^/kg, 10×10^6^/kg	Fludarabine (30mg/kg), Cyclophosphamide (300mg/kg)	No GvHD, CRS, or ICANS	ORR 73%, CR 64%
NCT04074746 (30)	MD Anderson	Phase I/II recruiting (n=30)	NKCE	AFM13 precomplexed with CB-derived NK cells	CD30-expressing R/R lymphoma	1×10^6^/kg, 1×10^7^/kg, 1×10^8^/kg	Fludarabine (30mg/kg), Cyclophosphamide (300mg/kg)	No GvHD, CRS, or ICANS. No DLT.	ORR 97%, CR 63%

Method: the website clinicaltrials.gov was accessed on 1/16/2023, and search terms “CAR+NK”, “Chimeric+antigen+NK”, “CAR+natural+killer”, and “Chimeric+antigen+natural+killer” were used to identify ongoing CAR NK-cell studies; each study was further reviewed and those using CB as the source were included in the table. Likewise, search terms “natural+killer+engager”, “NK+engager” were used to identify ongoing NKCE trials; studies that used precomplexed CB-derived NK-cells were included in the table. R/R, relapsed/refractory. NHL, non-Hodgkin lymphoma. N/A, not available; AML, acute myeloid leukemia; MM, multiple myeloma; GvHD, graft-versus-host disease; CRS, cytokine release syndrome; ICANS, immune effector cell-associated neurotoxicity syndrome; ORR, overall response rate; CR, complete response rate; DLT, dose-limiting toxicity.

### 3.3 CB-derived CAR NK-cells

CAR NK-cells have several advantages over CAR T-cells. First, they have a favorable safety profile due to the lack of TCR, with low risks of GvHD, cytokine release syndrome and neurotoxicity (26). Second, CAR NK-cells possess dual mechanisms of killing, through both CAR and innate receptors (27), which can potentially reduce the chance of CAR-targeted antigen escape. Compared with autologous CAR NK-cells, allogeneic CAR NK-cells also have advantages. First, “off-the-shelf” allogeneic products can significantly reduce the cost and increase availability. Second, as the activation of autologous CAR NK-cells is negatively affected by their innate inhibitory receptors (27); the use of allogeneic NK-cells can avoid the innate inhibition signal and enhance CAR NK activities.

Liu et al. reported the first-in-human trial of CAR-NK cells derived from unmatched or partially matched HLA cord blood in 2020 (26). The CAR-NK cells were transduced with a vector encoding anti-CD19 CAR, CD28 co-stimulatory domain, IL-15, and a safety switch of inducible caspase 9, and infused in 11 patients with B-cell lymphoma. No GvHD, CRS, or neurotoxicity was observed even at the highest dose; a response rate of 73% was reported, although the duration of response was unable to be assessed due to subsequent treatments. Of note, although CAR copies were still detectable in the peripheral blood after one year, on flow cytometry, CAR NK-cell rapidly declined after two weeks. Li et al. from the same group found that trogocytosis, a process involving transfer of tumor surface proteins (CD19) to CAR NK-cells upon anti-CD19 CAR/CD19 ligation, resulted in fratricide among CAR NK-cells and led to limited persistence (29). Co-infusion of KIR-based inhibitory CARs were able to counteract this process (29). Other ongoing CB-derived CAR NK-cell are summarized in **Table 2**.

The limited persistence of CAR NK-cells can also be an advantage from a safety standpoint. Therefore, CAR NK-cells may be an ideal tool for bridging to HCT. If a patient was ineligible for HCT, repeated infusions of CAR NK-cells might be needed to achieve long-term immune surveillance. However, previous studies using allogeneic unmodified NK cells have demonstrated that repeated NK-cell infusion could result in even shorter persistence (106). Therefore, clinical studies are needed to test whether repeated CAR NK-cell infusions are safe and effective.

### 3.4 Current challenges with CB-derived NK cell therapy

Autologous NK cells obtained from patients with cancer have suppressed effector function and poor clinical activities, likely due to the presence of “self” MHC molecules and NK cell exhaustion from cancer TME (121). Allogeneic NK cells provide an intriguing alternative despite still facing many challenges – some are intrinsic to the NK cell therapy; others are specific to the allogeneic nature.

First, in the absence of cytokine support, NK cells lack initial expansion after adoptive transfer (23). Although this can be improved by co-administration of IL-2, it is associated with increased toxicity (100, 122). In recent years, applying high intensity lymphodepleting chemotherapy prior to NK cell infusion has become a standard protocol, which creates a cytokine sink and leads to a surge of IL-15, facilitating NK cell expansion (104). However, the overall expansion is still poor especially in certain solid tumors (104).

Second, long-term persistence of functional allogeneic NK cells has not been established. On the other hand, it has been shown that CAR T-cells are able to form a stable clone persisting over 10 years, which is likely the key to long-term remissions in certain indolent cancers (123). Without long-term tumor immunosurveillance, a high relapse rate from NK cell therapy has been observed (124). Therefore, most clinical studies have only us`ed allogeneic NK cells as a bridging treatment to more definitive therapies such as HCT (26, 125). In recent years, a combination of cytokines were found to be able to induce NK-cell differentiation into memory-like NK cells, which have improved persistence to months in the matched-donor setting after HCT (126, 127). However, its use in the true allogeneic setting needs to be further explored (107). In another attempt, NK cells transduced with an IL-15 gene with the ability of endogenous IL-15 secretion have shown improved persistence to over a year (26, 128). Moreover, knockout of a negative regular of IL-15 signaling (cytokine checkpoint) can further improve NK cell persistence (129). However, more clinical studies are needed to confirm the functional status of persistent NK cells. Another factor leading to the short persistence is T-cell and host NK cell allorejection. To overcome this, knockout of HLA class I molecule and overexpression of inhibitory NK receptors have been proposed (130).

Third, as shown in tumor biopsy samples, NK cells have limited intra-tumoral infiltration or homing ability (24); moreover, NK cells are usually dysfunctional within the tumor due to its immunosuppressive TME (131). These factors likely lead to limited clinical activities of NK cell therapy in solid tumors. Strategies to enhance NK cell trafficking to tumor tissue and restore their function within TME are being developed in animal models, awaiting to be tested in the clinical setting (132).

Fourth, NK cells are subject to cryoinjury – cryopreserved and thawed post-expansion NK cells have reduced cytotoxicity which might become a hurdle for large-scale allogeneic NK cell production – although the significance of such impaired activity remains to be studied in the clinical setting (25).

## 4 CB-derived stem cell therapy

The most common application of banked CB is to use as a source for allogeneic HCT — around 500 CB-HCT are performed in the US each year (133). Although limited by the quantity as a single unit, CB hematopoietic stem cells possess a higher capability for self-renewal and proliferation than bone marrow stem cells (134). Moreover, the immune tolerant state and low frequency of alloreactive T cells in the graft have likely led to a lower incidence of GVHD and less restrictive HLA matching for successful engraftment (33, 135). Ever since CB-HCT was first performed in 1988 (5), several landmark studies have significantly improved the safety and efficacy of CB-HCT. For example, studies have shown that 1 or 2 allele mismatches did not impair engraftment, albeit a slightly increased risk of aGVHD and non-relapse mortality (32, 136); the development of double partially HLA-matched CB units (dCB) has expanded the use of CB-HCT to adults, where the cell dose in a single unit is frequently insufficient (137). Nevertheless, limited number of initial stem cells and slow engraftment remain the two biggest challenges in CB-HCT. Indeed, the current guideline from American Society for Transplantation and Cellular Therapy (ASTCT) recommends filtering out CB units with low total nucleated cell and CD34+ cell count as the initial steps for selection, irrespective of good HLA matching (138). Moreover, a study found that only 4% of CB units in the US CB bank inventory were suitable for a single unit CB-HCT (139). Therefore, techniques to improve CB *ex vivo* and *in vivo* expansion are needed to expand the use of CB-HCT.

With recent advancements in haploidentical HCT, which greatly expanded the donor source, the need for CB-HCT has been questioned due to its high cost and limitations. Indeed, the recently published prospective BMT CTN 1101 trial showed that compared to haploidentical HCT with post-transplantation cyclophosphamide, dCB-HCT had similar rates of GVHD, but a higher non-relapse mortality rate and lower overall survival, in even experienced CB transplant centers (35). However, CB-HCT still has its role in certain situations. First, about 10% patients in the US do not have a sibling, matched unrelated, or haploidentical donor, making CB the only feasible choice (140). Second, the BMT CTN 1101 trial allowed partially mismatched CB units and the median infused CD34+ cell dose was notably lower than the current ASTCT guideline (138); it is unknown if patients had fully matched CB units and received higher CB doses, their outcomes would be improved. Third, different strategies have been developed or are being developed to improve CB expansion and early hematopoietic recovery, which can further improve the safety of CB-HCT.

In addition to HCT, CB stem cells have also been studied in regenerative medicine, including organs that typically lack self-regenerative abilities, such as nerve, heart, or cirrhotic liver. However, a detailed discussion on this topic is beyond the scope of this review; we will refer reader to other updated review on this topic (141).

### 4.1 Strategies to improve *in vivo* expansion

To improve the *in vivo* expansion of CB after transplantation, several strategies have been tested in clinical trials. One attempt was to directly inject CB stem cells into the bone marrow (intrabone injection). It was first developed by Frassoni et al. with the hypothesis of reducing trapping in organs such as liver after intravenous injection, improving homing to the bone marrow, and lowering required cell dose for CB HCT (142). Multiple phase I/II studies have confirmed feasibility of this approach, with neutrophil engraftment time ranging from 17 to 23 days with lower than standard total nucleated cell (TNC) and CD34+ cell doses (142–148). In a retrospective study comparing intrabone injection with traditional dCB-HCT, the intrabone approach was associated with an improved neutrophil engraftment (23 vs 28 days) and a lower relapse rate (25 vs 29% at 2 years), despite receiving significantly lower cell doses (median TNC count 2.5 vs 3.9 × 10^7^/kg) (149). Therefore, intrabone injection is particularly useful when CB cell dose is insufficient and no alternative HCT approach is available. Phase III studies are needed to further investigate the efficacy of this approach.

Another method is to combine a small dose of CB with CD34-selected PB HSCs from a haploidentical donor. This haplo-cord approach resulted in a rapid early engraftment from the haploidentical donor, which was later replaced by the more durable engraftment of CB cells (150, 151). A retrospective study compared haplo-cord approach with haploidentical HCT with BM graft and post-transplantation cyclophosphamide, and found that the two approaches were associated with similar progression-free survival and overall survival, but the haplo-cord approach was associated with faster neutrophil engraftment and lower incidences of GvHD (152). However, the haplo-cord approach requires extra resources to obtain both CB and haploidentical donor cells; further, its efficacy needs to be confirmed in a prospective trial. Another approach to augment CB-HCT that has been developed recently was to infuse third-party granulocytes, with the goal to provide allogeneic MHC class I signaling, which would stimulate CD8+ T-cell proliferation to increase graft-versus-leukemia effects (153). In a proof-of-concept trial of 4 pediatric patients undergoing CB-HCT, the infusion of granulocytes led to more than 20-folds of CD8+ T-cell expansion (153). However, the safety of this approach, such as the occurrence of pre-engraftment syndrome, needs to be further studied.

### 4.2 Strategies to improve *ex vivo* expansion

Progress has been made in the past few years to improve the *ex vivo* expansion of CB through manipulation of culture conditions. These techniques have resulted in a higher stem cell dose at the time of infusion, with accumulating clinical studies demonstrating improved outcomes.

One strategy to optimize CB expansion is to co-culture with mesenchymal stem cells (MSCs), which can provide necessary microenvironment and cytokine support (154, 155). de Lima et al. showed that this approach led to a 12-fold increase in nucleated cells and a 30-fold increase in CD34+ stem cells, with the final product containing a median of 1.8×10^6^ CD34+ cells per kilogram (154). The improvement of initial cell dose translated into a significantly faster neutrophils engraftment than those undergoing single unit CB HCT (15 days vs 24 days) (154).

Another strategy to improve CB expansion is to add certain agents to the culture media (156). A variety of molecules, including Notch ligand (157, 158), copper chelation agents such as TEPA (159), vitamin B derivatives such as nicotinamide (160), aryl hydrocarbon receptor antagonists (AHRs) (161), and pyrimidoindole derivative UM171 (162), have shown improved expansion in clinical studies (**Table 3**). For example, nicotinamide can inhibit differentiation of hematopoietic stem cells; when added to CB culture media, an outgrowth of phenotypically primitive stem cells has been observed (163). A phase III clinical trial using nicotinamide expanded CB stem cells, known as the omidubicel, has shown significantly improved engraftment when compared with single or double-unit standard CB HCT (median neutrophil engraftment time 10 vs 20 days) (34). Omidubicel has also resulted in fewer bacterial and viral infections and less time in the hospital. Based on the trial results, omidubicel is currently seeking FDA approval for patients with hematologic malignancies in need of allo-HCT. However, it is important to note that these techniques require *ex vivo* manipulation of CB, which can lead to increased complexity and cost.

**Table 3:** Completed trials of *ex vivo* expansion of cord blood for hematopoietic stem cell transplantation.

Reference	Phase of trial	Expansion method	Expanded TNC, median, ×10^7^/kg	Expanded CD34+ cells, median, ×10^6^/kg	Conditioning and transplant	Neutrophils engraftment time, median (range), days	Platelet engraftment time, median (range), days	Infection outcome	Survival outcome
de Lima (154)	Phase I (n=31)	MSC	5.8	0.95	MAC	15 (9 to 42)	42 (15 to 62)	1-yr f/u: bacteria 9.7%, fungal 16.1%	1-yr OS: 32.2%
Mehta (155)	Phase I (n=27)	MSC	5.7	1.6	RIC	12 (1 to 28)	31 (9 to 25)	5 deaths due to infection	2-yr OS: 32%
Delaney (157)	Phase I (n=10)	Notch ligand	4.6	6	MAC, co-infusion of 1u unmanipulated CB	16 (7 to 34)	N.R.	N.R.	1-yr OS: 70%
Milano (158)	Phase II (n=15)	Notch ligand	5.8	5.2	MAC, non-HLA matched product, co-infusion with standard CB	19 (9 to 31)	35 (21 to 86)	N.R.	3-yr DFS: 86%
Stiff (159)	Phase II/III (n=101)	Copper chelator	3.1	0.14	MAC	21 (95%CI 18 to 24)	54 (95%CI 43 to 62)	5 deaths due to infection within 100 days	100-day OS: 84%
Horwitz (160)	Phase I/II (n=36)	Nicotinamide	4.9	6.3	MAC	11.5 (95%CI 9 to 14)	34 (95%CI 32 to 42)	5 deaths due to infection	2-yr OS: 51%
Horwitz (34)	Phase III RCT (n=125, 1:1 assigned)	Nicotinamide	4.7 vs 5.0	9.0 vs 0.3	MAC, omidubicel vs standard CB	10.0 (95%CI 8 to 13), vs control 20.5 (95%CI 18 to 24)	37.0 (95%CI 33 to 42), vs control 50 (95%CI 42 to 58)	Grade 2/3 infection 37% vs 57% in control within 100 days	210-day NRM: 11% vs 24%
Wagner (161)	Phase I/II (n=17)	SR-1	5	17.5	MAC, co-infusion of 1u unmanipulated CB	15 (6 to 30)	49 (28 to 136)	N.R.	1-yr OS: 55%
Cohen (162)	Phase I/II (n=22)	UM171	2.92	0.14	MAC	18 (12.5 to 20)	42 (35 to 47)	No infection related mortality	1-yr OS: 90%

TNC, total nucleated cells; MSC, mesenchymal stem cells; MAC, myeloablative conditioning; RIC, reduced intensity conditioning; f/u, follow up; OS, overall survival; N;R;, not reported; CB, cord blood; DFS, disease-free survival; RCT, randomized controlled trial; NRM, non-relapse mortality.

## 5 Cord-derived mesenchymal stem cells

Mesenchymal stem cells (MSCs) are multipotent perivascular progenitors that can differentiate into osteogenic, adipogenic, myogenic, or chondrogenic cells, making them an ideal resource for regenerative medicine. In addition, as part of the bone marrow niche microenvironment, MSCs possess immune modulatory properties by secreting cytokines, chemokines, and extracellular vesicles, rendering them great potential in treating inflammatory or autoimmune conditions (164). Although MSCs can be obtained from adult tissues such as the bone marrow or adipose tissue, CB and its compartments derived MSCs have advantages of ease in harvesting, increased stemness potential, and improved expansion (165). MSC therapies have consistently been proven safe, but clinical outcomes have been mixed and later phase studies are often disappointing despite efficacy signals in early phase studies. The discrepancy can be partially explained by MSC heterogeneity between donors, different manufacturing protocols for MSCs, and handling protocols after manufacture (166, 167). Moreover, due to short persistence of MSCs after infusion, repeated administrations are needed to achieve long-term effects. Despite these limitations, MSCs have been increasingly studied in regenerative medicine, inflammatory and autoimmune diseases including Covid-19. A detailed discussion on these topics is beyond the scope of this review, and we refer readers to other recently published review articles in this journal (168–170).MSCs have demonstrated two clinical applications in the field HCT. One is to promote *ex vivo* expansion and sustain *in vivo* hematopoiesis of HSCs, and the other is to prevent and treat GvHD given their immunomodulation ability. Several studies utilized co-infusion of CB-derived MSCs with allogeneic HSCs in patients with aplastic anemia – a condition known to be associated with poor graft function and a high risk of graft failure – in order to improve transplant outcomes (171–173). For example, Wang et al. showed that in 22 patients undergoing alloHCT for aplastic anemia who also received co-infusion of CB-derived MSCs, all achieved successful neutrophil engraftment in a median of 14 days; after 15 months’ follow-up, 21 patients were still alive and all maintained full donor chimerism (173).

MSCs have also been applied in GvHD. In the seminal study by Le Blanc et al. in 2004, bone marrow derived MSCs were first used in a patient with grade IV treatment-resistant acute GvHD and achieved great outcomes (36). Since the initial case report, numerous studies have been conducted to investigate various sources of MSCs in both acute and chronic GvHD. Although most studies were performed with BM-derived MSCs (174), a few studies have shown promising results from CB or its compartments derived MSCs. For example, in a phase I trial of 10 patients with *de novo* high-risk or steroid-refractory acute GvHD, Soder et al. showed that Wharton’s jelly-derived MSCs achieved an overall response and complete response rate of 70% and 40%, respectively (175). However, in a large phase III trial of 260 patients with steroid-refractory acute GvHD who were randomized to receive BM-derived MSCs *versus* placebo added to second-line therapy, there was no significant improvement of durable complete response (40). Therefore, more research is needed to fully understand the potential of these cells in the treatment of GvHD. CB-derived MSCs have also been tested to prevent GvHD (37, 38). In a phase II trial by Gao et al., 124 patients were randomized to receive monthly CB-derived MSCs or placebo for a maximum of 4 cycles starting at least 4 months after haploidentical alloHCT (38). They reported that the MSC group had significantly lower 2-year cumulative incidence of chronic GvHD than the placebo (27.4 vs 49.0%). Of note, the optimal timing of MSC infusion to prevent GvHD especially acute GvHD needs to be further investigated, as too early infusion might increase the risk of disease relapse (39).

## 6 Concluding remarks

With recent advancement in adoptive cell transfer, CB has demonstrated great and unique potential in various types of cellular therapy. As a source for allogeneic CAR T-cell therapy, it is possible to generate an HLA-homozygous CAR T-cell bank that matches most of the population, providing off-the-shelf CAR T-cell products with minimal GvH side effect and without genome editing. As a source for NK-cell therapy, recent early-phase studies have shown promising results with CAR NK-cells, as well as co-infusion of NK-cell engager and preactivated NK-cells. As a source for HCT, recent development in various techniques have greatly shorten the engraftment time resulting in improved safety. As a source of MSC, clinical studies for various autoimmune and inflammatory conditions such as GvHD are underway. However, most of the clinical studies are early phase; further developments are needed to facilitate more CB-derived cellular therapies into the market. Moreover, many of the CB-derived cellular products are regulated as advanced therapy medicinal products (ATMPs) both in the US and in the EU due to more-than-minimal or substantial biological manipulations of the CB cells. Although the regulatory frameworks were installed to ensure the safety and quality of cellular products, the complexity of regulatory requirements imposes significant challenges particularly for academic researchers who lack the resources to meet them.

## Author contributions

LM conceived the project. JW and LM reviewed the literature and drafted the article. All authors contributed to the article and approved the submitted version.
